# Nocturia at the Population Level in Poland: Prevalence, Bother, Quality of Life, and Treatment-Related Behavior

**DOI:** 10.3390/healthcare9050555

**Published:** 2021-05-10

**Authors:** Mikolaj Przydacz, Piotr Chlosta

**Affiliations:** Department of Urology, Jagiellonian University Medical College, 30-688 Krakow, Poland; piotr.chlosta@gmail.com

**Keywords:** Poland, epidemiology, population, nocturia, prevalence, bother, quality of life, Eastern Europe

## Abstract

*Background and Objectives:* The purpose of this study was to measure at the population level the prevalence, bother, effect on quality of life, and behavior associated with treatment for nocturia in a large representative cohort of Polish adults aged ≥40 years. *Materials and Methods:* Data were derived from LUTS POLAND, a computer-assisted telephone survey of urological health that mirrored the entire Polish population in urban and rural areas. *Results:* Six thousand persons completed the survey. The respondents were representative for age, sex, and place of residence. Nocturia was highly prevalent because 73.7% of all participants reported ≥1 nocturia episode, and 36.1% reported ≥2 nocturia episodes. We did not identify differences between urban and rural areas. Women were more often affected than men, and the prevalence of nocturia increased with age. More than one-third (29.7–45.3%) of respondents who reported nocturia were bothered by the symptom and, thereby, concerned about their urinary-specific quality of life. Notably, we found a statistically significant correlation between the frequency of nocturia and intensification of its bother (*p* < 0.001 for men and women). However, only about one-fourth (22.2–29.2%) of respondents with nocturia sought treatment, most of whom received treatment. *Conclusions:* Nocturia was highly prevalent and often bothersome with negative effects on quality of life of Polish adults aged ≥40 years. However, the percentage of treatment seeking was low. Combined with the fact that nocturia has multiple causes and consequences, including high morbidity and mortality, our findings revealed that nocturia was a significant public health issue. We need to develop strategies to specifically increase awareness about nocturia and provide greater healthcare and financial resources for persons with nocturia in Poland.

## 1. Introduction

The International Continence Society (ICS) defines nocturia as the number of times urine is passed during the main sleep period, with each urination followed by sleep or the intention to sleep [1]. Consequently, nocturia is one of the most bothersome lower urinary tract symptoms (LUTS) because repeated fragmentation of sleep causes daytime drowsiness and poor concentration. These conditions lead to further problems in personal and occupational functioning and impairments in physical and emotional health, finally shrinking overall well-being and quality of life [2].

Nocturia affects a high proportion of adults, and its prevalence has been investigated by several large population-based studies from various regions of the world, including Western Europe, North and South America, Asia, and Africa [3,4,5,6,7]. These studies often indicated that nocturia was the most prevalent LUTS; therefore, nocturia is likely a globally significant public health concern. Similarly, in a recent nationwide, prospective, population-representative, and cross-sectional study of LUTS in Poland, we determined that nocturia was the most prevalent symptom [8]. Notably, nocturia is usually multifactorial with particular relevance to heart disease, diabetes, kidney failure, or sleep problems. Thus, population estimates for nocturia are greatly needed to expedite formation of interdisciplinary foundations for national health programs, promote adequate allocation of government and healthcare systems resources, and increase public awareness. Further, persons reporting nocturia have a higher mortality rate, not only from an increased incidence of falls and fractures, but also from cardiovascular disease and early death [9]. Despite these facts, nocturia is often underestimated and underdiagnosed because most clinicians of different disciplines do not consider diagnosing and treating excessive nocturnal voiding as a serious concern for their patients [1].

Importantly, precise population-level data for nocturia are completely lacking for Central and Eastern Europe. To date, investigators have not performed any reliable large population-representative study that evaluated exclusively the prevalence, bother, and effect on quality of life of nocturia as defined by ICS. Moreover, because multiple factors may affect health and health-related behavior (e.g., culture, ethnicity), treatment behavior associated with nocturia in Poland may vary from behavior patterns reported for other countries or entities. Poland has a unique set of demographics (i.e., supra-ethnic uniformity, ≥99% of residents of Caucasian race, and ≥95% of residents of Polish identity) [10] that justify a comparison of Polish population estimates with estimates from less homogeneous populations [11]. Taking all into account, it is important to analyze more deeply and carefully nocturia based on the data from our population-based survey, LUTS POLAND [8]. Therefore, the aim of this study was to detail exclusively the prevalence, bother, effects on quality of life, and behavior related to treatment for nocturia in a large population-representative sample of persons aged ≥40 years living in all geographical regions of Poland. These estimates are needed to adequately guide Polish healthcare system, policy, and clinical practice.

## 2. Materials and Methods

This study was based on data from the LUTS POLAND survey; detailed descriptions of the concepts, study design, methodology, and data collection are published and reported only in brief in this paper [8]. LUTS POLAND was a population-based, cross-sectional survey of LUTS and overactive bladder (OAB). We interviewed a representative group of men and women, aged ≥40 years, from all geographical regions and areas of Poland, both urban and rural. The Jagiellonian University Medical College Ethics Committee approved this population-level investigation (1072.6120.160.2019). In addition, the study was registered with ClinicalTrials.gov (NCT04121936). In our initial analysis of LUTS POLAND, all symptoms were assessed equally with a standardized protocol based on the ICS definitions, supported by Likert-like scales [8]. In the current study, we analysed LUTS POLAND further with a focus on nocturia, including analyses based on additional questions that investigated the symptom more thoroughly.

Ipsos Poland collected data prospectively with computer-assisted telephone interviews; the study was validated first by pilot telephone surveys to assess cultural, linguistic, cognitive, and content integrity [12]. All participants were queried about nocturia (“Over the past month, how many times did you typically get up at night to urinate?”) [1,13], and respondents rated its occurrence and bother with Likert-like scales (occurrence: “none, one time, two times, three times, four times, five or more times”; bother: “not at all, a little bit, somewhat, quite a bit, a great deal, a very great deal”). During the interview, respondents also assessed how bladder problems affected their urinary-specific quality of life (assessment was based on the last question from the International Prostate Symptom Score, IPSS). Additionally, we analyzed nocturia-related questions from the OAB-V8, a validated screening tool for overactive bladder syndrome that includes nocturia. Both IPSS and OAB-V8 instruments are validated in Polish [14,15]. Eventually, participants were queried about their treatment-related behavior, particularly the seeking and receiving of treatment.

To create a target and representative group, we used the latest census and a sample matching technique [10,16]. Calculation of statistically appropriate sample size was based on the population age distribution and expected LUTS prevalence [17]. The survey sample was stratified before and after completing all responses to ensure representation for age, sex, and place of residence (both for geographical regions and type/size of places of living). Because one nightly void does not appear to be sufficiently disruptive to cause significant bother for most patients [18,19], and two nocturia episodes are proposed as a threshold for clinically significant nocturia [20], we estimated the nocturia prevalence with two thresholds: ≥1 and ≥2 nocturia episodes. We pre-specified this objective of the LUTS POLAND study in the statistical analysis plan, before initiating the survey.

Descriptive statistics were used for demographic variables and initial data evaluation. Continuous (numeric) variables (e.g., age, number of nocturia episodes) were subjected to the Kruskal–Wallis test, and categorical (containing a finite number of categories or distinct groups) variables (e.g., sex, type/size of place of living) were analyzed by the chi-squared test to evaluate differences in nocturia prevalence. Linear association between frequency and bother of nocturia was evaluated using Spearman’s rank correlation coefficient (r_S_; ranging from −1 indicating very strong negative association to + 1 indicating very strong positive association). A *p*-value < 0.05 was considered statistically significant. We used SPSS Statistics software (IBM, version 24.0, Armonk, NY, USA) for all data analyses.

## 3. Results

Overall, 6005 respondents from throughout Poland, representative for age, sex, and place of residence, participated in the survey. There were more women than men, and more respondents were living in urban than in rural areas.

### 3.1. The prevalence of Nocturia

**Figure 1:**
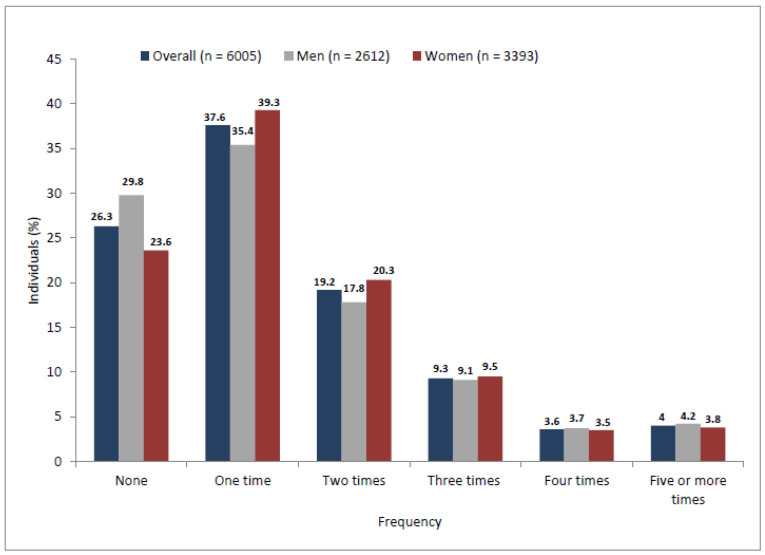
Prevalence of nocturia.

Nocturia was highly prevalent, and one occurrence of nocturia was the most often reported number of episodes (*n* = 2259, 37.6%, Figure 1). Notably, only about one-fourth of respondents denied any nocturnal voiding episodes (*n* = 1578, 26.3%). According to the threshold of ≥1 nocturia episode, the prevalence of nocturia was 73.7%. With the threshold of ≥2 episodes, the nocturia prevalence was 36.1%. Overall, nocturia was more prevalent in women than in men (*p* = 0.01 for ≥1 nocturia episode; *p* < 0.05 for ≥2 nocturia episodes, Figure 1). This observation was particularly relevant for respondents who reported one nocturia episode (Figure 1). With higher numbers of nocturia episodes, the difference in nocturia prevalence between men and women either decreased or the nocturia prevalence tended to be higher in men than in women (particularly for individuals who reported at least four nocturia episodes; not a statistically significant observation, *p* > 0.05).

Age group analysis of respondents who reported ≥1 nocturia episode showed higher prevalence of nocturia in women compared with men in all age groups (Table 1). However, with the threshold of ≥2 nocturia episodes, nocturia tended to be more prevalent in women compared with men in younger age groups, and more prevalent in men compared with women in older age groups (i.e., after 70 years of age; not a statistically significant observation, *p* > 0.05, Table 1).

**Figure 2:**
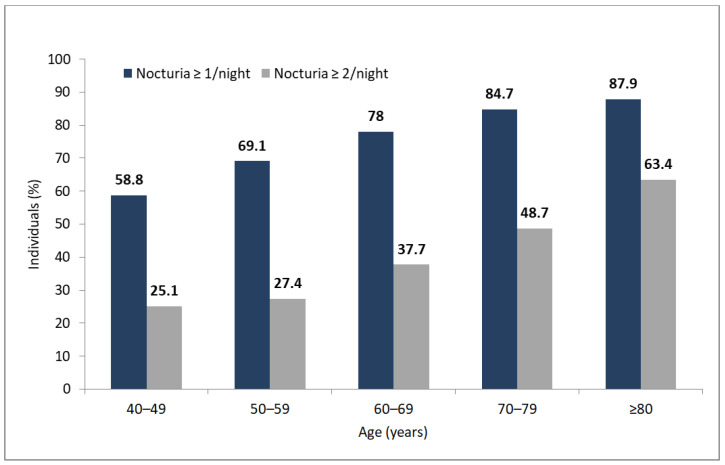
Prevalence of nocturia in age groups based on the two thresholds: nocturia ≥ 1 episode, and nocturia ≥ 2 episodes.

There was no difference in prevalence between urban and rural areas (for ≥1 nocturia episode: 73.2% vs. 74.5%, *p* > 0.05; for ≥2 nocturia episodes: 36.0% vs. 36.3%, *p* > 0.05). There was also no difference in nocturia prevalence across all geographical regions (voivodships) of Poland. In addition, we found that the prevalence of nocturia increased with age (*p* < 0.01). This age-dependent increase of nocturia prevalence was apparent with both thresholds, i.e., ≥1 and ≥2 nocturia episodes (Figure 2).

### 3.2. The Bother from Nocturia

**Figure 3:**
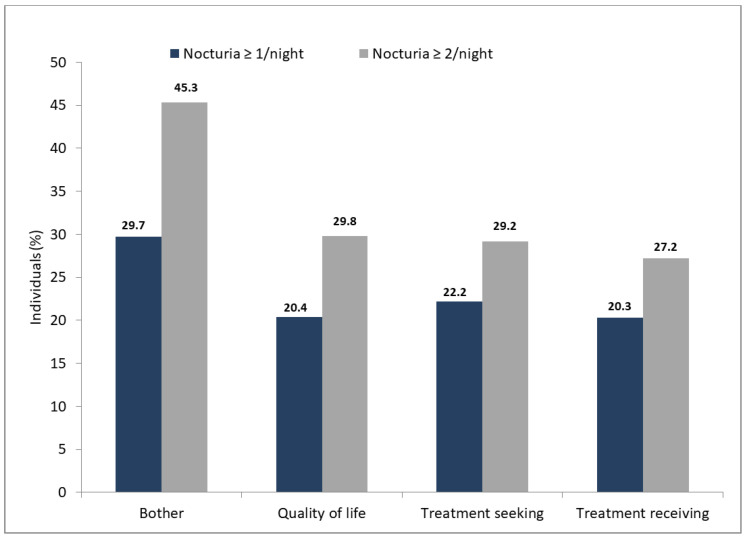
The bother of nocturia (“How bothered have you been by nighttime urination” with answers at least “quite a bit”), effect on quality of life (“If you were to spend the rest of your life with your urinary condition just the way it is now, how would you feel about that?” with answers at least “mixed”) and treatment-related behavior.

When asked, “How bothered have you been by nighttime urination?”, about one-third (29.7%, *n* = 1315) of the participants with at least one nocturia episode replied “quite a bit”, “a great deal”, or “a very great deal” (Figure 3). In a group of respondents with at least two episodes of nocturia, nearly half (45.3%, *n* = 982) of participants indicated that bother related to nocturia was “quite a bit”, “a great deal”, or “a very great deal”.

Similarly, for the question: “How bothered have you been by waking up at night because you had to urinate?”, one-third (30.3%, *n* = 1342) of the respondents with at least one nocturia episode replied “quite a bit”, “a great deal”, or “a very great deal”. The corresponding proportion for respondents with at least two episodes of nocturia was 45.2% (*n* = 981).

Overall, we found a statistically significant correlation between the frequency of nocturia and intensification of its bother for both sexes (r_S_ = 0.622 for men; r_S_ = 0.677 for women; *p* < 0.001 for men and women).

### 3.3. The Effect of Nocturia on Quality of Life

One-fifth (20.4%, *n* = 904) of respondents with ≥1 nocturia episode were prompted to respond ‘mixed’, ‘mostly dissatisfied’, ‘unhappy’, or ‘terrible’ when asked, “If you were to spend the rest of your life with your urinary condition just the way it is now, how would you feel about that?” (Figure 3). With a threshold of ≥2 nocturia episodes, about one-third (29.8%, *n* = 645) of the participants responded similarly. Regardless of the nocturia threshold, the negative effect of nocturia on quality of life was comparable between men and women.

### 3.4. Treatment-Related Behavior

Approximately one-fourth (22.2%, *n* = 982) of respondents with at least one nocturia episode pursued treatment for their bladder problems, and most of these individuals obtained treatment (20.3%, *n* = 900, Figure 3). In a group of persons who reported at least two nocturia episodes, one-third (29.2%, *n* = 634) sought treatment for their bladder issues, and, again, most of these persons received treatment (27.2%, *n* = 590, Figure 3). Although men tended to be more active than women in seeking treatment (nocturia ≥ 1: 23% vs. 21.5%; nocturia ≥ 2: 30.9% vs. 27.9%), this trend was not statistically significant. We did not identify disparities between treatment seeking/receiving, urban/rural areas, and geographical regions (voivodships) of Poland.

## 4. Discussion

The extension of the LUTS POLAND survey that we present in this paper is the first population-level analysis in Central or Eastern Europe that specifically addressed nocturia and quantified its prevalence, bother, effect on quality of life, and treatment-related behavior. Further, we used data from a one-country, nationally representative group of adults. We showed that nocturia was highly prevalent and bothersome. Although often nocturia had a negative effect on quality of life, treatment-seeking for this symptom was relatively uncommon.

The prevalence of nocturia has been investigated by a group of large population-level analyses. The Epidemiology Urinary Incontinence and Comorbidities (EPIC) study, a telephone survey in Canada, Germany, Italy, Sweden, and the UK (*n* = 19,165), showed that nocturia (≥1 episode) was the most common symptom with more women affected than men (54.5% women, 48.6% men) [4]. In South America, the Brazil LUTS, a telephone interview conducted in five major cities of Brazil (*n* = 5184), also showed that nocturia (≥2 episodes) was highly prevalent with higher prevalence in women (32.4%) than in men (27.1%) [5]. Our finding (*n* = 6005) of nocturia in 73.7% (≥1 nocturia episode) and 36.1% (≥2 nocturia episodes) of adults aged ≥40, which affected more women than men, appears broadly comparable with data from the other population-based studies. Therefore, nocturia is likely to be a significant global concern because surveys conducted in various regions of the world uniformly have revealed a high overall prevalence of nocturia. Environmental factors certainly differ, and population genetic backgrounds often differ, between the different places where nocturia investigations have been performed. Thus, it seems that the high prevalence of nocturia is quite independent of environmental or genetic effects. Because we conducted our study on a representative pool of adults from a highly homogeneous population, our results may be considered somewhat universal emphasis of the global scope of nocturia. Further, because of worldwide population aging, the burden of nocturia will increase because, as we found, the prevalence increases with age.

Nocturia was often bothersome with negative effect on quality of life. Further, we found a significant relationship between the frequency of nocturia and intensification of its bother, i.e., the more frequent the occurrence of nocturia, the more severe the bother. Nocturia-related poor sleep is a key factor responsible for decreased quality of life. Nocturia increases the number of wake-ups, and nocturnal voids are an independent predictor of both self-reported insomnia (75% increased risk) and reduced sleep quality (71% increased risk) [21]. Repeated fragmentation of sleep due to nocturia affects daytime functioning, that, in turn, can cause fatigue, cognitive impairment, mood alterations, embarrassment, poor self-esteem, and even some symptoms of depression [22,23]. Breyer et al. found that waking up at night to void increased from 1.2 to 20.24 the likelihood of reporting depression [24]. Because nocturia increases susceptibility to different bothersome conditions, it becomes a significant public health issue. Individuals with nocturia tend to have low productivity at work, and they use more healthcare services compared with individuals free of nocturia [25]. These are a major problem for working people ≤65 years old. Diminished work activity causes reduced work efficiency and economic loss for society. Therefore, nocturia should not be dismissed; instead, adequate public health attention should be given for this symptom. Population estimates are especially important to uncover the real-life impact of nocturia.

The level of treatment seeking for bladder problems was low for people who reported nocturia. This observation is particularly worrisome because there is growing recognition that nocturia is a symptom with a wide-ranging pathophysiology, including blood pressure changes, cardiac dysfunction, renal disorders, fluid shift into the lower limbs, polyuria, diabetes, sleep apnea, insomnia, pharmacotherapy, and polypharmacy [26]. Therefore, nocturia may be a red flag for one or more serious medical conditions that may significantly decrease survival. The Nagahama study, a longitudinal population cohort study from Japan, showed that nocturia was associated with high mortality. Mortality increased in a “dose-dependent” manner, with the nocturnal voiding frequency hazard ratio of 1.46 for 1 time, 1.85 for 2 times, and 2.06 for 3 or more times compared with 0 times (*p* < 0.001) [27]. A systematic review and meta-analysis by Pesonen et al. presented similar findings and demonstrated that an approximately 1.3-fold increased risk of death was probably associated with nocturia [28]. All these findings suggest that there is an ongoing need to improve public awareness of nocturia in Poland and other countries. Education should also target different types of healthcare professionals, particularly non-urologists. We should especially underline the role of primary care physicians, who can often screen for nocturia and initiate simple diagnostics with sometimes noninvasive treatment modalities. In addition, our results indicated that there is probably a need for higher healthcare and financial resources for nocturia in Poland because individuals with nocturia should obtain a patient-oriented multidisciplinary diagnosis with adequate, often combined, therapy. Importantly, treatments for nocturia include optimization of reported comorbidities, lifestyle changes, behavioral therapies, pharmacotherapy (with particular interest in desmopressin for nocturnal polyuria), and sometimes even surgery (e.g., bladder augmentation in patients with low bladder capacity). The ICS recommends a holistic multidisciplinary approach to nocturia [26].

We did not find any differences between urban and rural areas in treatment seeking and treatment receiving among persons who reported nocturia. Before starting the LUTS POLAND survey, we expected that people living in urban areas would be more active in seeking help for their bladder problems relative to people from rural areas. We based our speculation on a study by Branowitzer, who reported that people from rural areas in Poland were more hesitant to admit or discuss their health issues [29]. However, data from that study are archaic. Poland joined the European Union in 2004 and, since then, several large health improvement programs in Polish rural areas have been instituted [30]. Although longitudinal studies that would investigate the effects of these programs on population health were not conducted, our study supports the concept that health differences between Polish urban and rural areas are dissolving. Further, with increasing population density in Poland, we also need to acknowledge that Polish urban and rural areas have started to overlap [30].

This study has several limitations, including the nature of data capture and data self-reporting. Some respondents might not have been fully open or honest with cold-callings. In addition, we did not ask about concomitant medical disorders. However, during a telephone survey, without clinical verification, it would have been difficult to reliably establish from a self-reporting participant the existence of other medical conditions. Coyne et al. described this significant information bias of population-based self-reported data [31]. In addition, we did not collect data concerning barriers to seeking healthcare.

## 5. Conclusions

This investigation was the first nationwide, population-representative epidemiological study of nocturia to be performed in a Central or Eastern European country. Nocturia was highly prevalent among Polish adults aged ≥40 years, and more women were affected than men. Nocturia was often bothersome with negative impact on quality of life. However, only about 25% of affected individuals sought treatment. Taking into account multiple causes and consequences of nocturia together with its high population-level prevalence, we have established the fact that nocturia is a significant public health issue. We need to develop strategies to specifically increase the population awareness about nocturia in Poland. In addition, higher healthcare resources are needed to better diagnose and treat nocturia patients.

## Figures and Tables

**Table 1:** Prevalence of nocturia in men and women based on the two thresholds (nocturia ≥ 1/night; nocturia ≥ 2/night) in different age groups.

Age Groups	Men	Women
Nocturia ≥ 1/night		
40–49	357 (56.5%)	358 (61.3%)
50–59	416 (66.2%)	599 (71.2%)
60–69	588 (74.7%)	881 (80.4%)
70–79	355 (83.1%)	575 (85.7%)
≥80	118 (85.5%)	180 (89.6%)
Nocturia ≥ 2/night		
40–49	145 (22.9%)	160 (27.4%)
50–59	155 (24.7%)	248 (29.5%)
60–69	290 (36.8%)	420 (38.3%)
70–79	218 (51.1%)	317 (47.2%)
≥80	92 (66.7%)	123 (61.2%)

## Data Availability

Data is contained within the article.
